# Comparison between adult and foetal adnexa derived equine post-natal mesenchymal stem cells

**DOI:** 10.1186/s12917-019-2023-5

**Published:** 2019-08-02

**Authors:** B. Merlo, G. Teti, A. Lanci, J. Burk, E. Mazzotti, M. Falconi, E. Iacono

**Affiliations:** 1 Department of Veterinary Medical Sciences, University of Bologna, via Tolara di Sopra 50, 40064 Ozzano Emilia, BO Italy; 2 Department for Biomedical and Neuromotor Sciences, University of Bologna, Bologna, Italy; 3 Saxon Incubator for Clinical Translation, University of Leipzig, Leipzig, Germany; 4 Equine Clinic (Surgery), Justus Liebig University Giessen, Giessen, Germany; 5 Department of Comparative Biomedical Sciences, University of Teramo, Teramo, Italy; 6 Health Science and Technologies Interdepartmental Center for Industrial Research (HST-ICIR), University of Bologna, Bologna, Italy

**Keywords:** Horse, Mesenchymal stem cells, Adult, foetal adnexa, Transmission electron microscopy

## Abstract

**Background:**

Little is known about the differences among adult and foetal equine mesenchymal stem cells (MSCs), and no data exist about their comparative ultrastructural morphology. The aim of this study was to describe and compare characteristics, immune properties, and ultrastructural morphology of equine adult (bone marrow: BM, and adipose tissue: AT) and foetal adnexa derived (umbilical cord blood: UCB, and Wharton’s jelly: WJ) MSCs.

**Results:**

No differences were observed in proliferation during the first 3 passages. While migration ability was similar among cells, foetal MSCs showed a higher adhesion ability, forming smaller spheroids after hanging drop culture (*P* < 0.05). All MSCs differentiated toward adipogenic, chondrogenic and osteogenic lineages, only tenogenic differentiation was less evident for WJ-MSCs. Data obtained by PCR confirmed MHC1 expression and lack of MHC2 expression in all four cell types. Foetal adnexa MSCs were positive for genes specific for anti-inflammatory and angiogenic factors (IL6, IL8, ILβ1) and WJ-MSCs were the only positive for OCT4 pluripotency gene. At immunofluorescence all cells expressed typical mesenchymal markers (α-SMA, N-cadherin), except for BM-MSCs, which did not express N-cadherin. By transmission electron microscopy, it was observed that WJ-MSCs had a higher (*P* < 0.05) number of microvesicles compared to adult MSCs, and UCB-MSCs showed more microvesicles than BM-MSCs (*P* < 0.05). AT-MSCs had a lower number of mitochondria than WJ-MSCs (*P* < 0.05), and mitochondrial area was higher for WJ-MSCs compared to UCB and AT-MSCs (*P* < 0.05).

**Conclusions:**

Results demonstrate that MSCs from adult and foetal tissues have different characteristics, and foetal MSCs, particularly WJ derived ones, seem to have some charactestics that warrant further investigation into potential advantages for clinical application.

## Background

Mesenchymal stem cells (MSCs), also known as multipotent stromal cells or mesenchymal progenitor cells, are of increasing interest in the regenerative medicine field. Populations of MSCs can be relatively easily isolated from tissues that differ both developmentally (e.g., foetal versus adult) and anatomically (e.g., bone marrow versus adipose tissue). Despite the common characterization and clinical applicability potential for all the different sources of MSCs, there are qualitative and quantitative differences in terms of their isolation efficiency and in vitro manipulation performance, as well as their efficacy in animal models and clinical studies, which have been highlighted both in human [1–5] and animals [6–11]. Diversity in cell manipulation, including the isolation and culture protocols applied, in addition to inherent heterogeneity in the samples related to donor, may have an impact on the quality and quantity of isolated cells. Therefore, issues concerning ease of isolation, cell yield, and donor site complications suggest that certain sources may be more favorable than others for a specific clinical application.

In the horse, MSCs from bone marrow (BM) possessed the highest in vitro osteogenic potential compared to adipose tissue (AT) and umbilical cord blood (UCB) and tissue (UCT) as indicated by osteogenic gene expression and mineral deposition [6]. Burk et al. (2013) confirmed the same observation but noticed that, in contrast, the highest levels of chondrogenic differentiation were observed in UCB- and UCT-MSCs [7]. On the other hand, AT-MSCs showed the highest expression of tendon extracellular matrix proteins and tendon differentiation markers [10]. When comparing BM to amnion derived MSCs, the placental cells showed a more rapid growth and clonogenic capability and a faster osteogenic differentiation [8]. In vivo application for treatment of horse tendon and ligament injuries of BM and amniotic membrane MSCs not only confirmed the advantage to administer allogeneic amniotic membrane MSCs when needed, before any ultrasonographic change occurs within the injured tendon and ligament, but also the lower re-injury rate observed after amniotic MSCs treatment let suppose that their implantation is more efficacious compared to BM-MSCs implantation [9].

Despite the investigations, still little is known about the differences among adult and foetal adnexa derived MSCs, and no data exist about their comparative ultrastructural morphology. Since different foetal adnexa derived equine MSCs showed ultrastructural differences [12], the aim of this study was to describe and compare expression of markers related to characterization and paracrine activity, and ultrastructural morphology of equine post-natal adult (BM and AT) and foetal adnexa derived (UCB and Wharton’s jelly-WJ) MSCs.

## Results

### Cell isolation and calculation of cell doubling times

Adherent mononuclear cells were characterized by a homogeneous elongated fibroblast-like morphology. Undifferentiated cells of different sources were passaged up to three times; no changes in cell morphology were observed throughout the culture period.

Considering data from 3 passages, no differences (*P* > 0.05) were observed in CDs (cell doublings) of different cell types (8.6 ± 0.4 vs. 9.0 ± 1.4 vs. 9.6 ± 0.9 vs. 9.9 ± 1.1 for BM, AT, UCB and WJ respectively). Mean DT (doubling time) was similar (*P* > 0.05) among groups (3.6 ± 1.4 days vs. 2.2 ± 1. 1 days vs 2.4 ± 1.3 days vs. 2.7 ± 0.9 days for BM, AT, UCB and WJ respectively).

Comparing data from single passages of the same cell type, no statistically significant differences were found in DTs (*P* > 0.05).

### Adhesion and migration assays

**Fig. 1:**
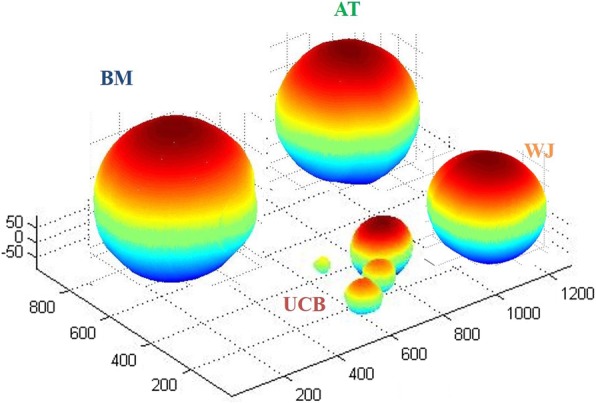
Adhesion assay results. Spheroids derived from equine bone marrow (BM), adipose tissue (AT), Wharton’s jelly (WJ) and umbilical cord blood (UCB) mesenchymal stem cells after 24 h hanging drop culture. Spheroids were reconstructed and visualized from a single projection using ReViSP. Measure unit: pixel

Both foetal and adult MSCs formed spheroids when cultured in hanging drops (Fig. 1). Average volume of the spheroids formed by WJ and UCB-MSCs was significantly lower (*P* < 0.05) as compared to BM and AT-MSCs, demonstrating a higher cell-cell adhesion capacity for foetal adnexa derived MSCs than adult MSCs.

Probably due to the large variation observed for AT-MSCs, average percentage of migration, observed by scratch test, was similar (*P* > 0.05) between cell types (WJ-MSCs 43.0 ± 7.7%, UCB-MSCs 28.4 ± 5.0%, BM-MSCs: 25.0 ± 10.2%, AT-MSCs 24.5 ± 21.4%) when analysed by one-way ANOVA. Comparison of groups using a Student T-test, revealed a higher (*P* < 0.05) migration rate for WJ-MSCs than for UCB and BM-MSCs, but not compared to AT-MSCs (*P* > 0.05), as could be expected due to the large variation observed in this group.

### In vitro differentiation

**Fig. 2:**
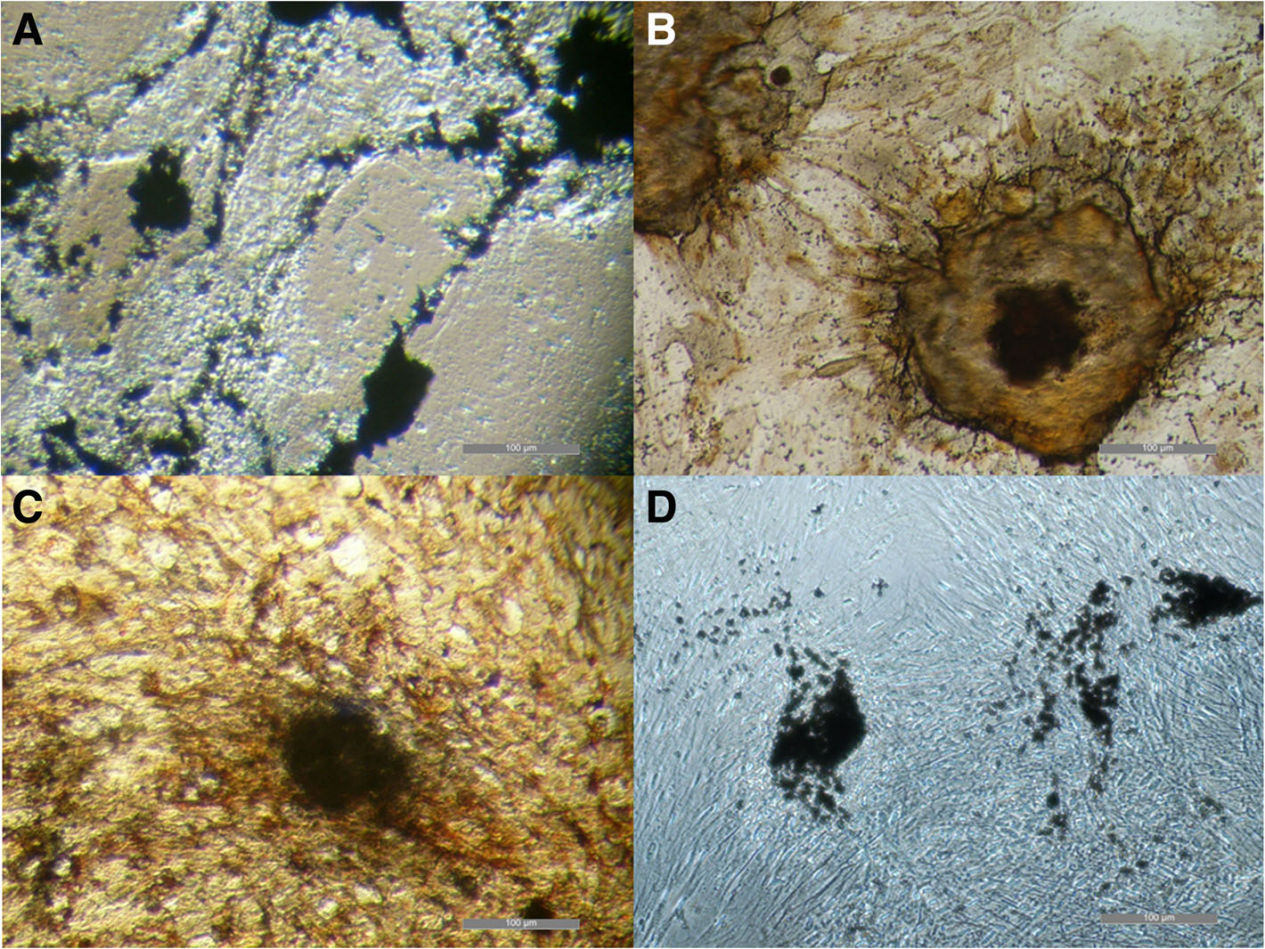
Equine mesenchymal stem cells (MSCs) osteogenic differentiation. Von Kossa staining of extensive extracellular mineral deposition in MSCs derived from umbilical cord blood (**a**) (bar: 100 μm), Wharton’s jelly (**b**) (bar: 100 μm), bone marrow (**c**) (bar: 100 μm) and adipose tissue (**d**) (bar: 100 μm)

**Fig. 3:**
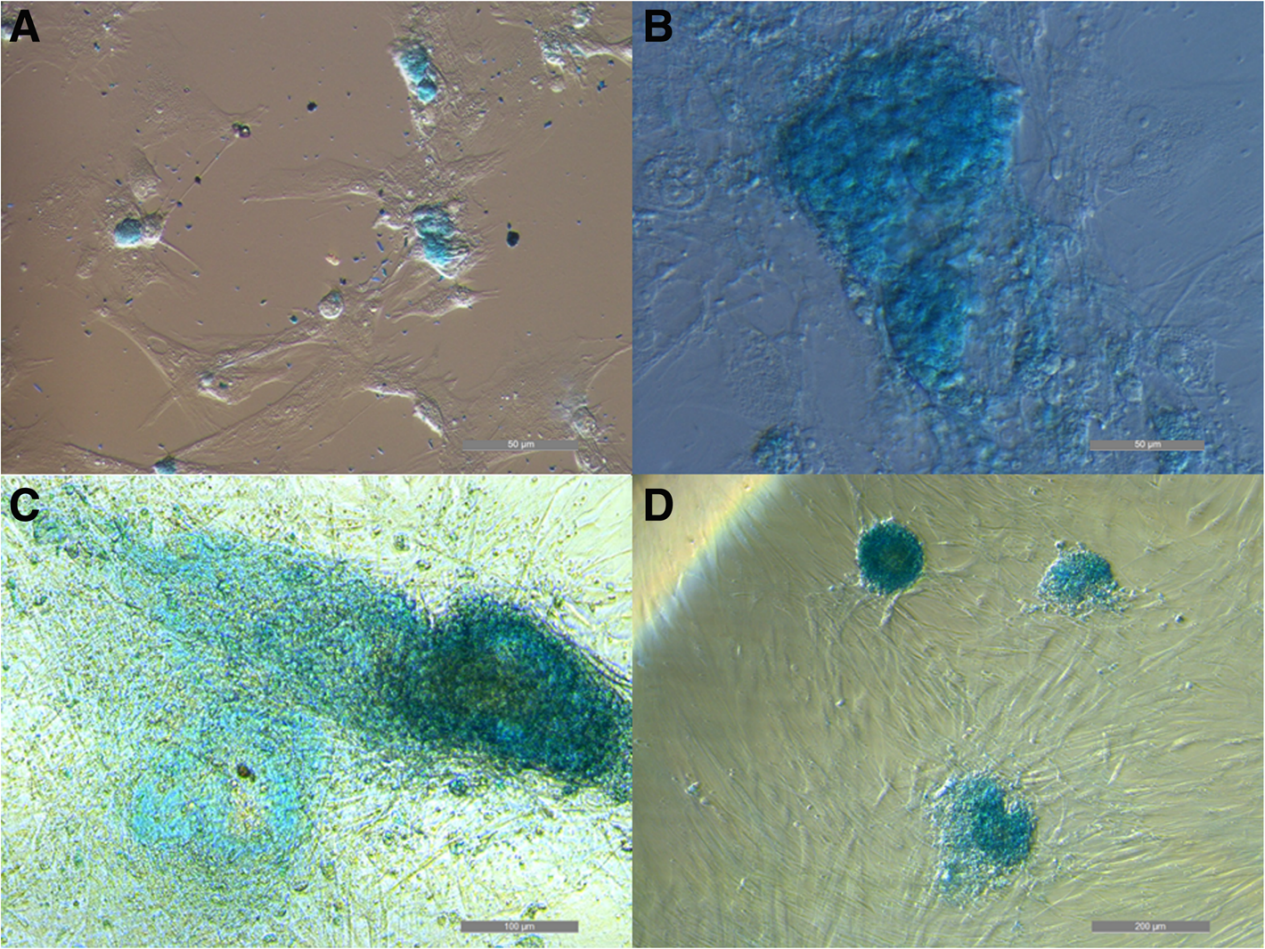
Equine mesenchymal stem cells (MSCs) chondrogenic differentiation. Alcian Blue staining of glycosaminoglycans in cartilage matrix in MSCs derived from umbilical cord blood (**a**) (bar: 50 μm), Wharton’s jelly (**b**) (bar: 50 μm), bone marrow (**c**) (bar: 100 μm) and adipose tissue (**d**) (bar: 200 μm)

**Fig. 4:**
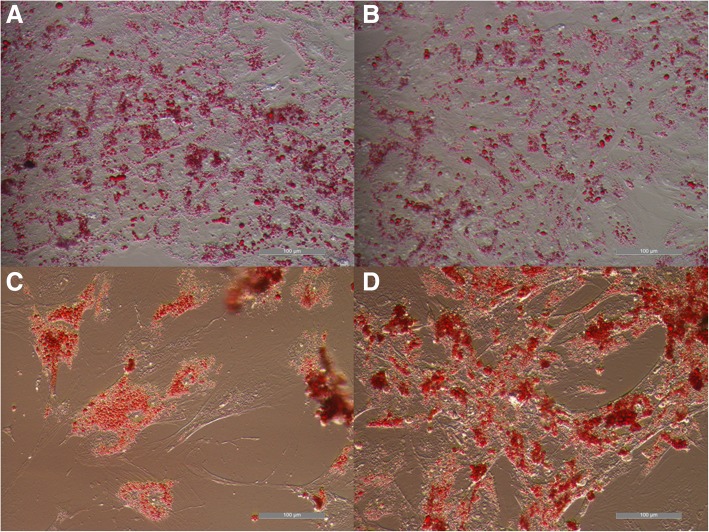
Mesenchymal stem cells (MSCs) adipogenic differentiation. Oil red O staining of extensive intracellular lipid droplet accumulation in MSCs derived from umbilical cord blood (**a**) (bar: 100 μm), Wharton’s jelly (**b**) (bar: 100 μm), bone marrow (**c**) (bar: 100 μm) and adipose tissue (**d**) (bar: 100 μm)

**Fig. 5:**
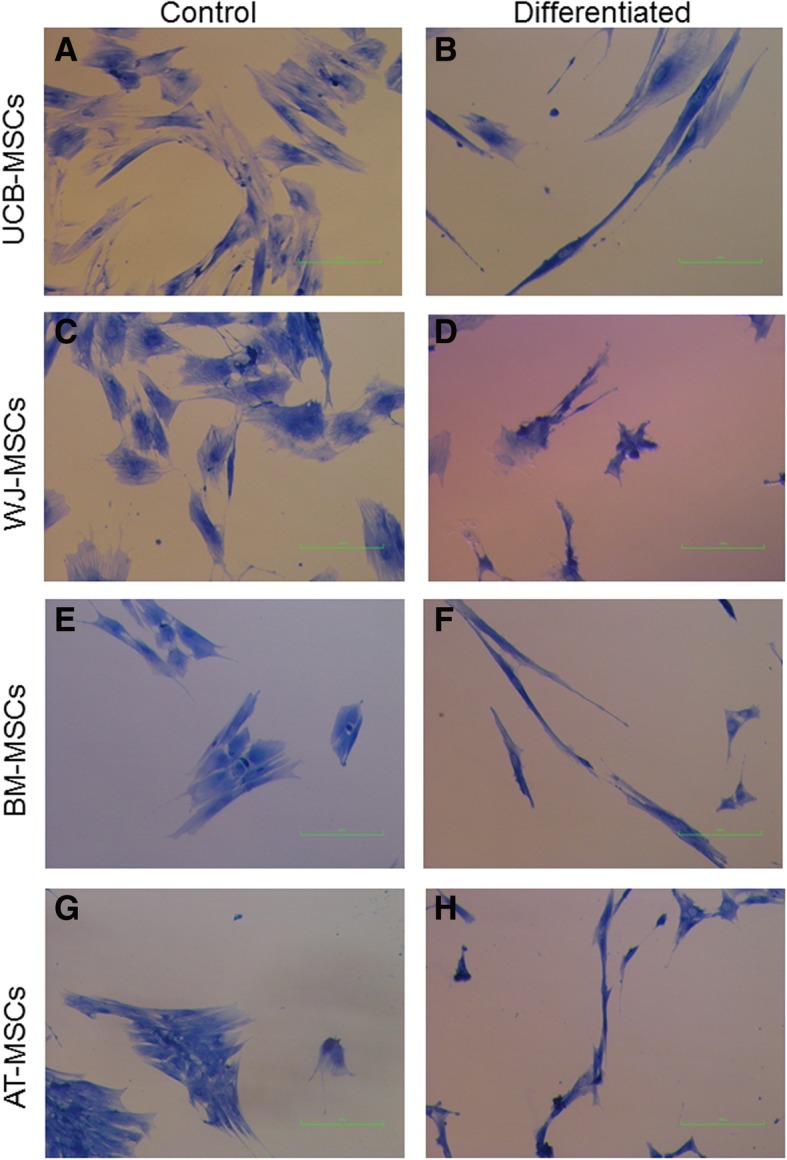
Equine mesenchymal stem cells (MSCs) tenogenic differentiation. Staining with Aniline Blue method of umbilical cord blood (UCB)-MSCs control cells (**a**) (bar: 100 μm) and differentiated cells (**b**) (bar: 100 μm), Wharton’s jelly (WJ)-MSCs control cells (**c**) (bar: 100 μm) and differentiated cells (**d**) (bar: 100 μm), bone marrow (BM)-MSCs control cells (**e**) (bar: 100 μm) and differentiated cells (**f**) (bar: 100 μm) and adipose tissue (AT)-MSCs control cells (**g**) (bar: 100 μm) and differentiated cells (**h**) (bar: 100 μm)

All cell types were able to differentiate toward osteogenic (Fig. 2), chondrogenic (Fig. 3), adipogenic direction (Fig. 4). Subjective observation could not find obvious differences among groups, but it is interesting to report that, even if cultured in monolayer for chondrogenic differentiation, UCB-MSCs tended to form a mass of differentiated cells in the dish. For this reason it was possible to get images only of the few cells still in mono-layer (Fig. 3a). It could suggest that UCB-MSCs have a very good potential for chondrogenic differentiation. For tenogenic differentiation (Fig. 5) WJ-MSCs showed poor morphology changes when compared to the other cell lines.

### Immunofluorescence (IF)

**Fig. 6:**
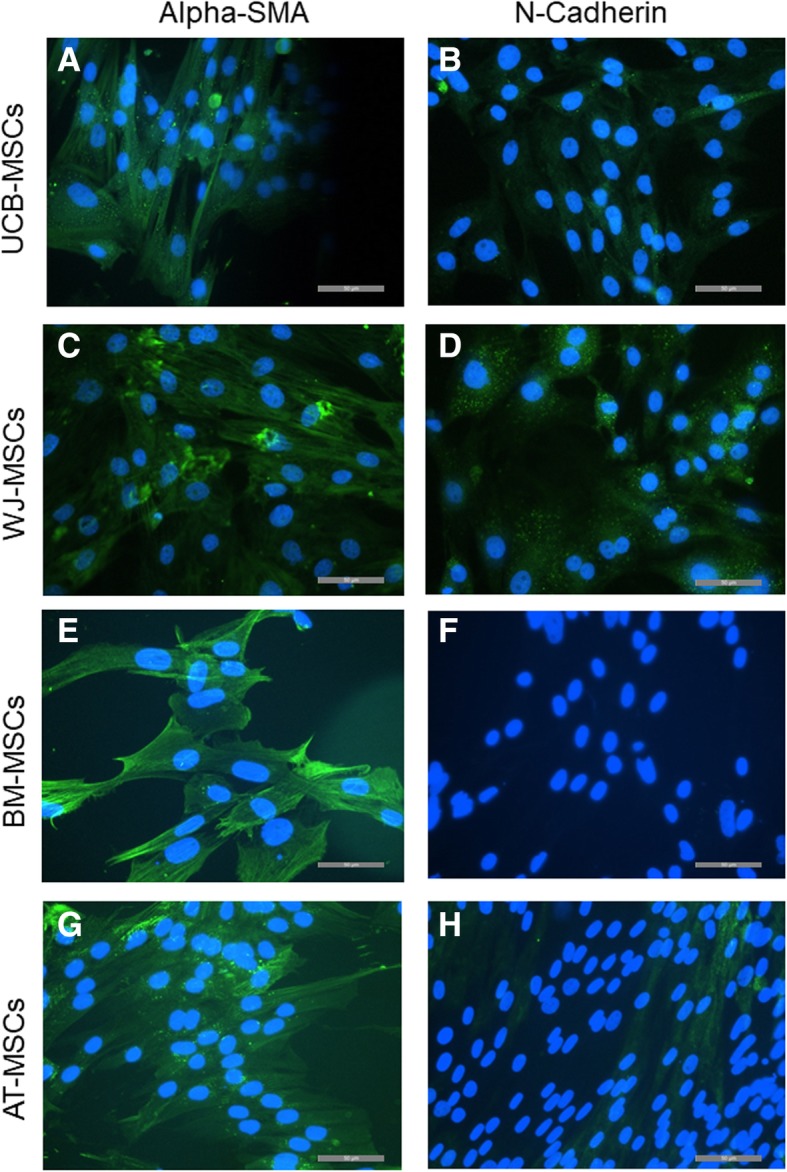
Photomicrographs of immunostaining of umbilical cord blood (UCB), Wharton’s jelly (WJ), bone marrow (BM) and adipose tissue (AT)-MSCs (mesenchymal stem cells). Expression of the mesenchymal marker α-SMA in the UCB (**a**) (bar: 50 μm), WJ (**c**) (bar: 50 μm), BM (**e**) (bar: 50 μm) and AT-MSCs (**g**) (bar: 50 μm). Expression of the mesenchymal marker N-Cadherin in the UCB (**b**) (bar: 50 μm), WJ (**d**) (bar: 50 μm), BM (**f**) (bar: 50 μm) and AT-MSCs (**h**) (bar: 50 μm). Green is FITC from each target marker and blue is nuclei staining with Hoechst 33342 (UCB and WJ-MSCs) or DAPI (BM and AT-MSCs)

Both adult and foetal cell types clearly expressed the mesodermal marker α-SMA (α-smooth muscle actin, Fig. 6a, c, e, g). On the contrary, BM-MSCs did not express the mesenchymal marker N-Cadherin while UCB, WJ and AT-MSCs did (Fig. 6b, d, f, h).

### Reverse transcriptase-polymerase chain reaction (RT-PCR)

Qualitative data (presence/absence) obtained by PCR are reported in Table 1. All cell types were positive for MSC marker CD90, while AT-MSCs were negative for CD73. All cells were negative for hematopoietic markers CD34 and CD45, except for WJ-MSCSs, that were positive for CD34. MHC1 was positive and MHC2 negative for all cell types. Only WJ and UCB-MSCs were positive for IL6 and IL8, and WJ-MSCs also for ILβ1. IL-4, TNFα and INFγ were not constitutively expressed in any cell type. All cells were negative for pluripotency markers except for WJ-MSCs that were positive for OCT4.

**Table 1:** Results obtained by PCR running on equine foetal adnexa derived and adult mesenchymal stem cells (MSCs). Qualitative data (presence/absence) are presented, grouped in categories

Primers	WJ-MSCs	UCB-MSCs	BM-MSCs	AT-MSCs
Mesenchymal markers				
CD90	+	+	+	+
CD73	+	+	+	–
Hematopoietic markers				
CD34	+	–	–	–
CD45	–	–	–	–
Major histocompatibility complex markers				
MHC1	+	+	+	+
MHC2	–	–	–	–
Pro- and anti-inflammatory citokines				
TNFα	–	–	–	–
IL8	+	+	–	–
INFγ	–	–	–	–
IL4	–	–	–	–
ILβ1	+	–	–	–
IL6	+	+	–	–
Pluripotency markers				
OCT4	+	–	–	–
NANOG	–	–	–	–
SOX2	–	–	–	–

*WJ* Wharton’s jelly, *UCB* umbilical cord blood, *BM* bone marrow, *AT* adipose tissue. +: positive; −: negative

### Transmission Electron microscopy (TEM) and morphometric analysis

**Fig. 7:**
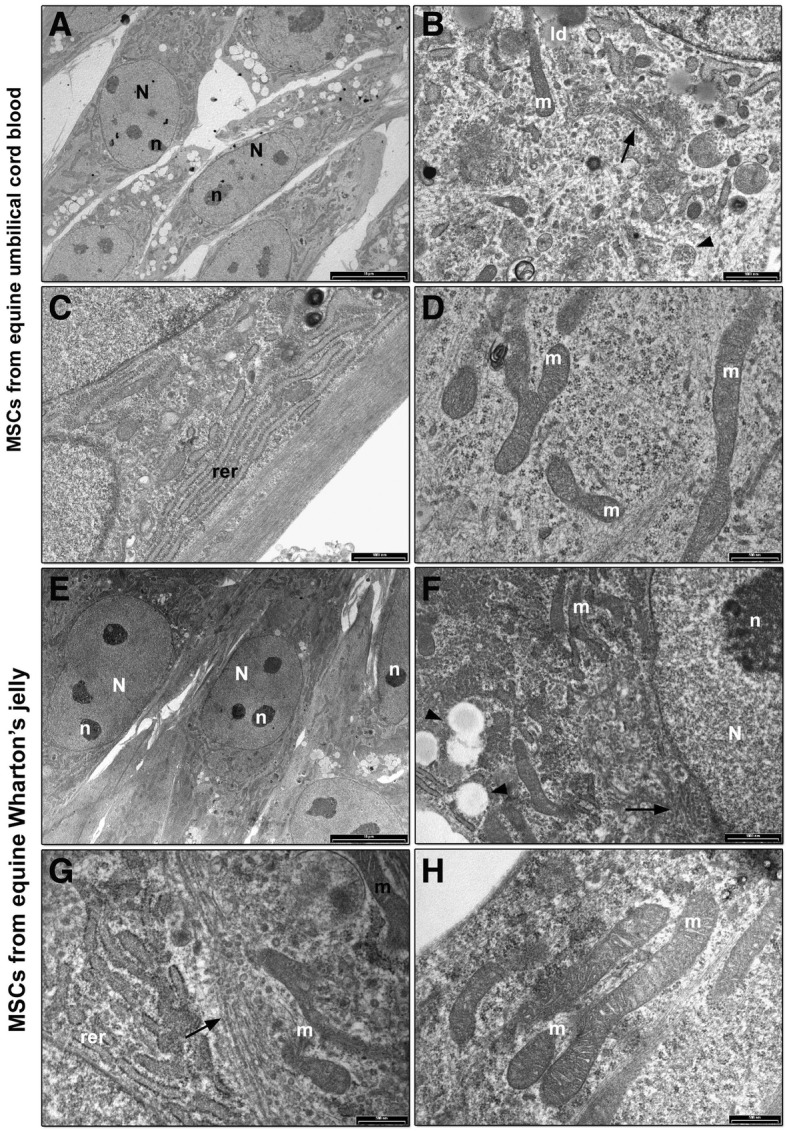
TEM analysis of equine foetal adnexa derived mesenchymal stem cells (MSCs). Umbilical cord blood MSCs (**a**) Cells show a fibroblast like morphology. Nucleus (N) and dark and dense nucleoli (n) are well detected (bar: 10 μm); **b** Golgi complex (black arrow), lipid droplets (li) and multivesicular bodies (black arrowhead) are observed in the cytoplasm (bar: 1 μm); **c** A well-developed RER, with long and narrow membranes, localized in the cytoplasm (bar: 1 μm); **d** Long and narrow mitochondria (m), with dense matrix and thin cristae, are observed in the cytoplasm (bar: 500 nm). Wharton’s jelly MSCs (**e**) low magnification image showing a cluster of MSCs with a spindle morphology. Nucleus (N) and dark and dense nucleoli (n) are easily detected (bar: 10 μm); **f** RER (rer), Golgi apparatus (black arrow), mitochondria (m) and lipid droplets (black arrow) were observed in the cytoplasm (bar: 1 μm); **g** detail of the cytoplasm showing a well-developed RER (rer) and Golgi complex (black arrow) surrounded by several vesicles and mitochondria (m) (bar: 500 nm); **h** Long and narrow mitochondria (m), with dense matrix and thin cristae, are observed in the cytoplasm (bar: 500 nm)

UCB-MSCs showed a fibroblast shape morphology (Fig. 7a) with the nucleus and nucleoli well detected. In the cytoplasm several organelles, such as Golgi apparatus, surrounded by several vesicles (Fig. 7b), a widespread rough endoplasmic reticulum (RER) (Fig. 7c) and narrow mitochondria, with dense matrix and thin cristae, (Fig. 7d) were detected. Lipid droplets (Fig. 7a) and multivesicular bodies (Fig. 7b) were also observed in the cytoplasm.

WJ-MSCs appeared with a spindle like morphology (Fig. 7e). Nucleus and compact and thick nucleoli were easily detected (Fig. 7e). At higher magnification, regular RER, lipid droplets and Golgi apparatus, closed around by several vesicles were observed (Fig. 7f and g). Mitochondria appeared scattered in the cytoplasm (Fig. 7h).

**Fig. 8:**
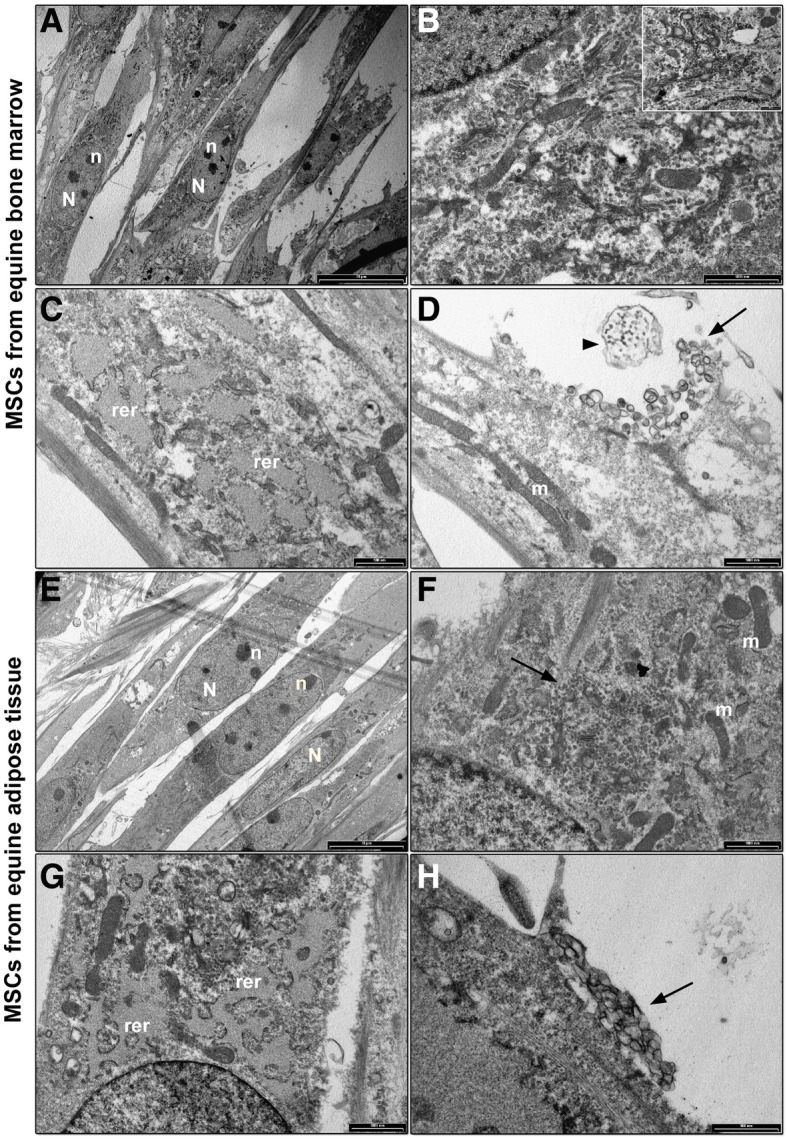
TEM analysis of equine adult mesenchymal stem cells (MSCs). Bone marrow MSCs (**a**) Cells show a fibroblast like morphology. Nucleus (N) and dark and dense nucleoli (n) are well detected (bar: 20 μm); **b** A well-developed Golgi complex surrounded by several vesicles and multivesicular bodies (white square; bar: 500 nm) are observed in the cytoplasm (bar: 1 μm); **c** Extended RER (rer), characterized by dilated cisternae, lacking of ribosomes, is shown in the cytoplasm (bar: 500 nm); **d** Long and narrow mitochondria (m), with dense matrix and thin cristae, are observed in the cytoplasm. Extracellular vesicles (black arrow) and exosomes (black arrowhead) are detected in the extracellular environment (bar: 1 μm). Adipose tissue MSCs (**e**) low magnification image showing a cluster of MSCs with a spindle morphology, nucleus (N) and dark and dense nucleoli (n) (bar: 10 μm); **f** Golgi apparatus (black arrow) and mitochondria (m) were observed in the cytoplasm (bar: 1 μm); **g** detail of the cytoplasm showing an extended RER (rer) characterized by dilated cisternae almost empty of ribosomes (bar: 1 μm); (H) (bar: 500 nm); **h** Aggregated extracellular vesicles (black arrow) are localized nearby cell membrane (bar: 500 nm)

BM-MSCs showed a fibroblast shape morphology (Fig. 8a) with nucleus and dense nucleoli easily detectable. Golgi apparatus, surrounded by several vesicles (Fig. 8b), scattered extensively in the cytoplasm. RER with dilated cisternae was observed (Fig. 8c), characterized by membranes almost lacking ribosomes. Mitochondria were detected in each cell section (Fig. 8c). Several extracellular vesicles and exosomes were observed in the extracellular membrane surface (Fig. 8d).

AT-MSCs showed a fibroblast like morphology (Fig. 8e) with nuclei and nucleoli well detected. A scattered Golgi complex, surrounded by vesicles (Fig. 8f) was observed. Mitochondria were present in the cell (Fig. 8f). The RER is localized in one end of the cell and it is characterized by enlarged membrane, almost free from ribosomes (Fig. 8g). Several extracellular vesicles were detected on membrane surface (Fig. 8h).

**Fig. 9:**
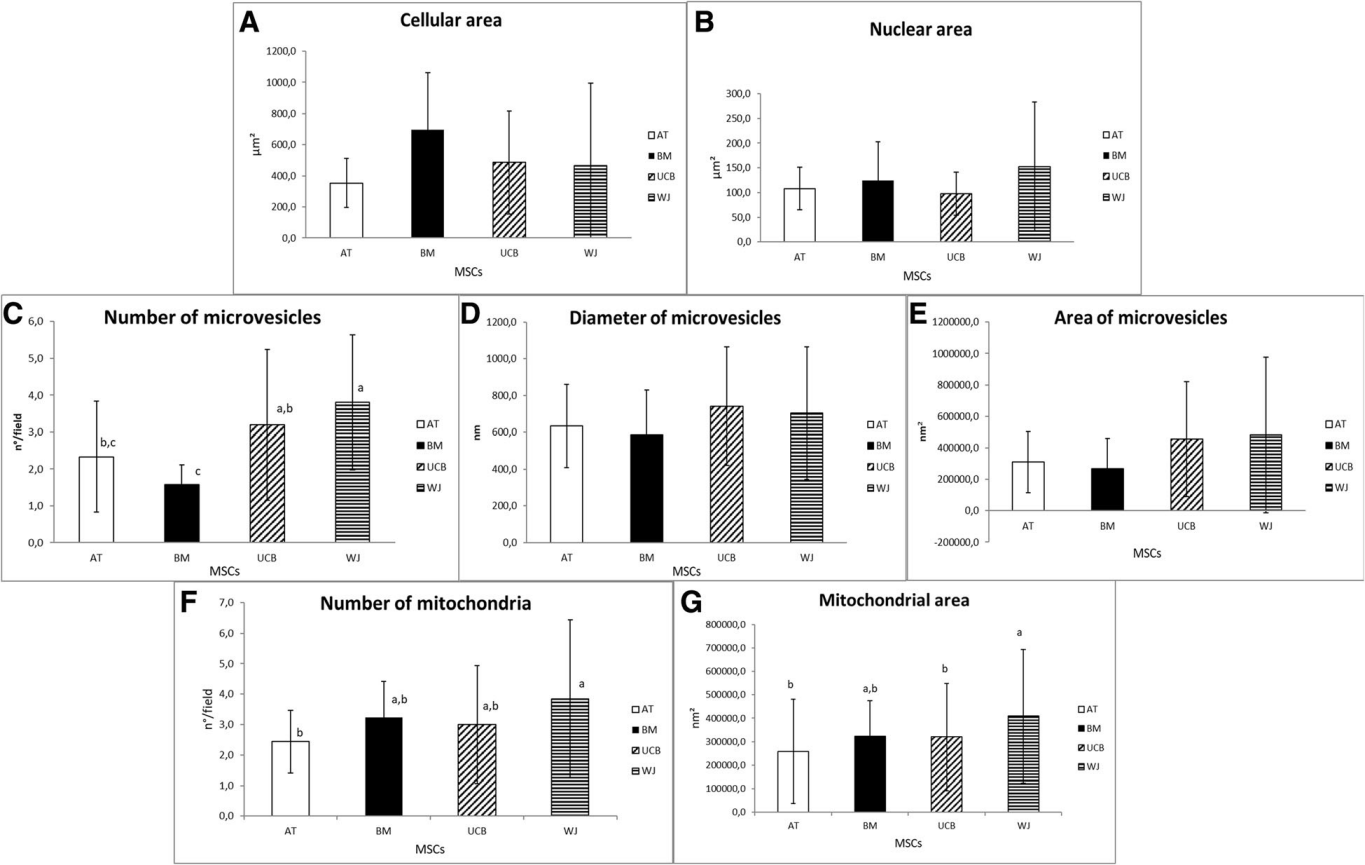
Morphometric analysis of equine adult and foetal adnexa mesenchymal stem cells (MSCs). Histograms represent means and standard deviations for (**a**) cellular area, **b** nuclear area, **c** number of microvesicles, **d** diameter of microvesicles, **e** area of microvesicles, **f** Golgi’s vesicles number, **g** Golgi’s vesicles diameter, **h** number of mitochondria, **i** area of mitochondria. Different letters mean significant differences (*P* < 0.05)

Total results from morphometric analysis are summarized in Fig. 9. The number of microvesicle was significantly higher (*P* < 0.05) for WJ-MSCs compared to adult MSCs and for UCB-MSCs compared to BM-MSCs (Fig. 9c). Also the number and area of mitochondria were different, since AT-MSCs had a lower number of mitochondria than WJ-MSCs (*P* < 0.05) (Fig. 9f) and WJ-MSCs showed a higher (*P* < 0.05) mitochondrial area compared to UCB and AT-MSCs (Fig. 9g).

## Discussion

In this study, MSCs from foetal adnexa (UCB and WJ) and adult tissues (BM and AT) were isolated and many features were compared: proliferation, migration, spheroids formation, differentiation capacity, expression of relevant markers and ultrastructural features. All isolated cells showed the typical mesenchymal morphology. Both foetal adnexa and adult MSCs were isolated, cultivated with standard protocols and they did not show differences for CD and DT. However, in this study, we considered only the first three passages of culture, in fact it has been already demonstrated that WJ had a shorter DT than UCB when analysing data derived from a longer period of culture (8 passages) [13]. In this study, only 3 passages were considered since for clinical application only early passages are usually used to avoid any effect of cellular senescence or transformation.

Migration potential of MSCs is considered important for their integration into the host tissue during therapeutic applications [14]. In the present study, WJ-MSCs showed a higher migration activity compared to UCB and BM-MSCs, but not compared to AT-MSCs, due to the high variability observed among samples in that group. The higher migration potential could suggest that WJ-MSCs graft integration in vivo may be enhanced.

At P3 all cells were differentiated to evaluate their osteogenic, chondrogenic, adipogenic and tenogenic potential. While morphological changes and differences after stainings showed similar differentiation ability of both foetal adnexa derived and adult MSCs toward osteogenic, chondrogenic and adipogenic lineages, tenogenic differentiation was less evident for WJ-MSCs as compared to the other MSCs. Since it is the first time that equine WJ-MSCs are differentiated toward tendon with the protocol used, it is still not clear if the difference is due to a not suitable tenogenic induction protocol or because these cells need a longer stimulation period.

In the present study, the cell-cell adhesion capability was measured by the in vitro spheroid formation potential using the hanging drop method. As already demonstrated, adhesion ability can be related to the chondrogenic differentiation potential [15–17]. Determining the spheroid volume (a smaller volume is related to a higher cell-cell adhesion ability), it was evident that foetal adnexa derived MSCs had a higher adhesion ability than adult cells, being able to form smaller spheroids. Cells derived from UCB created multiple small spheroids and were not able to give origin to a single spheroid per drop.

Immunofluorescence investigation showed a positive expression of α-SMA in all cell types, a protein expressed by cells of the mesodermal lineage [18]. However, BM-MSCs did not express N-Cadherin, another mesenchymal marker [18]. It can be speculated that BM-MSCs loose N-Cadherin expression during culture, since in another study regarding immunophenotypic characterization of equine BM-MSCs, it was observed that N-cadherin-positive cells were present at the second passage, whereas at the fifth passage these cells were not detected [19].

Equine MSCs from all tested sources expressed the genes coding for positive marker antigens and did not express genes coding for the negative marker as defined by the International Society for Cellular Therapy [20]. As previously observed [12], equine WJ-MSCs were positive for CD34. As recently demonstrated, the expression of CD34 seems to depend on the environment, on the in vitro culture cell passages and on the cell source [21, 22], supporting that the lack of CD34 marker can not be considered an essential requirement of a stem cell. AT-MSCs were negative for CD73, but as already demonstrated by flow cytometry, CD73 is rarely expressed by most equine MSCs [22–26]. Our results are based only on positive/negative gene expression but not on relative quantification of gene expression or in protein expression by flow cytometry or other techniques, which means that surface marker expression may differ from qualitative gene expression.

Foetal cells were unique in that they expressed some interleukins (IL8 and IL6 for both WJ and UCB-MSCs, ILβ1 for WJ-MSCs only). All the cells were negative for TNFα, IL4 and INFγ, cytokines that may be produced only after in vitro stimulation [27]. ILβ1, IL6 and IL8 are important mediators of the inflammatory response, involved in a variety of cellular activities, including cell proliferation, differentiation, apoptosis, chemotaxis, angiogenesis and haematopoiesis [28]. These factors are involved in the complex interaction between MSCs and the tissue microenvironment as well as production of membrane vesicles, containing molecules such as short peptides, proteins, lipids, and various forms of RNAs [29]. These findings encourage investigation in potential advantages of foetal adnexa derived MSCs for therapy.

As for SOX2 and NANOG, OCT4 is a typical marker of embryonic pluripotent cells [30–34] and it should not be expressed by MSCs. However, its expression by WJ-MSCs confirms their intermediate characteristics between adult and embryonic stem cells [35]. Although the OCT4 primer used in the present study was chosen from a research in which also immunofluorescent localisation of Oct4 protein was made on equine embryos [36], it has been demonstrated in human, mouse and pig that Oct4 has different variants [37–39] and that detection of Oct4 expression by RT-PCR could be prone to artifacts generated by pseudogene transcripts [40]. No specific research on equine Oct4 is available, therefore western blotting could be used in future work to further support our conclusions in the present study.

TEM morphological analysis of MSCs showed ultrastructural details connected with the different origin of cells. A fibroblast shape morphology, dense nucleoli, a widespread Golgi apparatus and well developed RER are morphological features linked to the mesenchymal phenotype [41, 42]. Golgi complex and RER are strictly connected with a high protein synthesis and high metabolic rate [41, 42]. The elevated secreting ability of these cells is further underlined either by the presence of several extracellular vesicles and exosomes, on cell surface, and multivesicular bodies, inside the cells, which are released in the extracellular microenvironment and act as paracrine factors [42]. Foetal adnexa MSCs, in particular WJ derived ones, showed a larger amount of microvesicles in the cytoplasm compared to adult MSCs. The presence of a constitutive autophagy has been demonstrated as a cytoprotective and cellular quality control mechanisms to balance protein and organelle turnover, crucial for the maintenance of stemness and for a number of differentiation processes [43, 44]. Furthermore, the therapeutic potential of mesenchymal stem cell-derived microvesicles has been largely demonstrated [45], which could confer a potential advantage to foetal adnexa MSCs for therapeutic use, even though these results are still preliminary.

Equine MSCs presented few mitochondria in each cell, with a long and narrow morphology. In all samples, mitochondria showed a very long and narrow shape, with a dense matrix and thin cristae, in agreement with in vitro cultured stem cells [46]. During stem cell derivation, a shift from orthodox mitochondria, with a characteristic light matrix and thin cristae, to condensed mitochondria, with a dense matrix and swollen cristae, has been described and it is thought to be connected with a decrease of membrane potential, increased oxygen and ADP/ATP ratio [46]. In detail, WJ-MSCs, with a high number of mitochondria and the larger mean mitochondrial area of 0,40 μm^2^, reached the highest value suggesting a higher energy requirements [47, 48] for WJ-MSCs needed for Golgi complex, protein synthesis and cell expansion.

## Conclusion

Taking together all the results, they warrant further investigation of therapeutic potential of foetal adnexa derived equine MSCs, particularly WJ-MSCs. The tenogenic differentiation ability of WJ-MSCs remains to be elucidated. While reduced tenogenic potential may be inconvenient, their therapeutic use in case of tendon injuries should be further investigated, since different factors play a role in therapeutic application beyond differentiation ability.

## Methods

Chemicals were purchased from Sigma-Aldrich (Milan, Italy), and laboratory plastic ware from Sarstedt Inc. (Verona. Italy), unless otherwise stated.

### Study design

Three samples for each tissue type were used. Cell were isolated, then doubling time was calculated from passage 0 (P0) to P3. At P3 all cells lines underwent adhesion and migration assay, tri-lineage in vitro differentiation plus tenogenic differentiation, immunofluorescence for α-SMA and N-cadherin, RT-PCR for different genes, and comparative ultrastructural morphology was investigated by TEM.

### Sample collection

UCB and WJ samples were recovered from foetal adnexa immediately after parturition of three healthy mares (Standardbred, 6–12 years old), housed at the Department of Veterinary Medical Sciences, University of Bologna, for attended delivery. Written informed consent from owners was obtained to collect samples for research purposes.

BM was collected from three experimental horses (Haflinger breed, 3 years old) at the Veterinary Teaching Hospital, Department for Horses, University of Leipzig, Germany. Bone marrow was collected from sedated horses according to standard surgical procedures. The sternal region was prepared aseptically, local anesthesia was applied, the sternum was punctured with an 11 G bone marrow aspiration needle and a sample was aspirated into a heparinized syringe. Horses were then euthanized for unrelated reasons and subcutaneous adipose tissue was collected via skin incision from the supragluteal region. Tendon samples, to induce the tenogenic differentiation in a transwell coculture system, were aseptically collected from the superficial digital flexor tendons of equine distal limbs collected at an abattoir, and immediately frozen at − 80 °C.

### Cell isolation and calculation of cell doubling time

Samples were stored in DPBS (Dulbecco’s Phosphate Buffered Solution) supplemented with antibiotics (100 IU/mL penicillin and 100 μg/mL streptomycin), at 4 °C, until processing. In the lab, cells were isolated as previously described [7, 13]. Briefly, mononuclear cells were separated from UCB and BM by standard density gradient centrifugation using a polysaccharide solution (Ficoll-Paque Premium, GE Healthcare). AT was minced and digested in a collagenase I solution (Invitrogen) (0,8 mg/ml) at 37 °C for 4 h; WJ was minced and digested in DPBS containing 0.1% collagenase (w/v) (GIBCO®, Invitrogen Corporation, Carlsbad, California, USA) by incubation at 37 °C for 20–30 min. Isolated BM and AT cells were seeded into culture flasks containing low concentration glucose (1 g/L) Dulbecco’s modified Eagle medium (DMEM) (Invitrogen), supplemented with 20% foetal bovine serum (FBS) (Sigma Aldrich), 100 IU/mL penicillin, 0.1 mg/mL streptomycin (1% penicillin–streptomycin) and 0.05 mg/mL gentamycin, and incubated in a 5% CO_2_ humidified atmosphere at 37.0 °C. UCB and WJ isolated cells were seeded in culture flasks containing DMEM-F12 Glutamax® (Gibco) supplemented with 10% (v/v) FBS (Gibco, Waltham, MA, USA) and antibiotics (100 IU/mL penicillin and 100 μg/mL streptomycin). Primary cells were plated in a 25 cm^2^ flask in culture medium and incubated in a 5% CO_2_ humidified atmosphere at 38.5 °C.

At 80–90% of confluence, cells were dissociated by 0.25% trypsin, counted and cryopreserved in case of BM and AT cells. Passage 1 (P1) cells were then plated at the concentration of 5 × 10^3^ cells/cm^2^, and so on until P3, culturing all cell lines in DMEM-F12 + antibiotics + 10% FBS. DT and CDs and cell culture time (CT) were calculated from hemocytometer counts for each passage according to the following two formulae [49]:

$$ \mathrm{CD}=\ln\ \left(\mathrm{Nf}/\mathrm{Ni}\right)/\ln\ (2) $$

$$ \mathrm{DT}=\mathrm{CT}/\mathrm{CD} $$

where Nf and Ni are the final and initial number of cells, respectively.

### Adhesion and migration assays

In order to define differences between adult and foetal adnexa derived MSCs, spheroid formation and migration test were performed at P3. For adhesion assay, cells were cultured in ‘hanging drops’ (5000 cells/drop) for 24 h. That method provides information about the direct cell-cell adhesion architecture found in normal tissues, differently from the cell-substratum adhesion, performed on monolayer cultures adherent to rigid substrates. Images were acquired by a Nikon Eclipse TE 2000-U inverted microscope. Spheroid areas were determined using ImageJ software Version 1.6. Starting from the binary masks obtained by Image J, the volume of each spheroid was computed using ReViSP [50], a software specifically designed to accurately estimate the volume of spheroids and to render an image of their 3D surface.

To assess cell migration potential, a scratch assay was carried out. Cells (4.8 × 10^4^) were seeded on 35 mm petri dishes and cultured until confluence in the same conditions as previously described. Scratches were made using 1 mL pipette tips, washed with medium and allowed to grow for additional 24 h. Immediately after the scratch and at the end of the culture, cells were observed under an inverted light microscope (Eclipse TE 2000u, Nikon Instruments Spa, Florence, Italy) and photographed in the same area (marked in the plate) by CCD camera (Visicam 3.0, VWR International Srl, Milan, Italy; DS-Fi2, Nikon Instruments Spa, Florence, Italy). Gap distance of the wound was measured using Image J software (Version 1.48 s; National Institutes of Health, USA). The migration percentages were calculated using the following formula:

[(distance at time 0–distance at 24 h)*100]/distance at time 0.

### In vitro differentiation

At P3, in vitro differentiation potential of cells monolayers towards osteogenic, adipogenic, chondrogenic and tenogenic lineages was studied. Cells (5000 cells /cm^2^) were cultured under specific induction media. Adipogenic medium: DMEM/F12 plus antibiotics + 15% (v/v) rabbit serum + 1 μmol/l dexamethasone (removed after 6 days) + 0.5 mmol/l IBMX (3-isobutyl-1-methylxanthine) (removed after 3 days), 10 μg/ml insulin, 0.2 mmol/l indomethacin. Chondrogenic medium: DMEM/F12 plus antibiotics + 1% (v/v) FBS + 6.25 μg/ml insulin + 50 nM AA2P (2-phospho-L-ascorbic acid trisodium salt), 0.1 μmol/l dexamethasone, 10 ng/ml hTGFβ1 (human transforming growth factor β1). Osteogenic medium: DMEM/F12 plus antibiotics + 10% (v/v) FBS + 10 mmol/l β-glycerophosphate + 0.1 μmol/l dexamethasone + 50 μmol/l AA2P. Tenogenic differentiation was induced as described by Lovati et al. (2012) [51]. Briefly, tendon fragments of 2 to 3 mm^3^ were seeded on the upper membrane (pore size of 0.4 μm) of a transwell chamber (Corning Costar, Cambridge, MA, USA) and co-cultured with 500 MSCs /cm^2^ seeded on the bottom of 6-well culture plates. 3 ml of serum-free medium were added to cover both the upper tendon pieces and the lower monolayer of MSCs.

Control samples consisted in MSCs cultured for the same period of time in DMEM/F12 plus 2% (v/v) FBS.

To assess differentiation, cells were fixed with 4% (w/v) paraformaldehyde, and then stained. Oil Red O (0.3% (v/v) in 60% (v/v) isopropanol) was used to evaluate formation of neutral lipid vacuoles after 10 days of adipogenic differentiation. Chondrogenic and osteogenic differentiation were assessed after 21 days of culture in induction media by using 1% (w/v) Alcian Blue in 3% (v/v) acetic acid solution and Von Kossa (1% (w/v) silver nitrate in water), respectively. Tenogenic differentiation was assessed after 15 days of culture using 2% (w/v) Aniline Blue in water.

Cells were observed under an inverted light microscope (Eclipse TE 2000u, Nikon Instruments Spa, Florence, Italy) and photographed by CCD camera (DS-Fi2, Nikon Instruments Spa, Florence, Italy).

### IF

Cells at P3 were fixed with 4% paraformaldehyde, then washed in phosphate buffer (PB). Cells were permebilized in 0.5% (v/v) Triton-X100 for 15 min and then blocked in goat serum (10%) for 1 h and incubated overnight with primary antibodies (rabbit polyclonal α-SMA 1:500 (Gene tex, Milan, Italy); rabbit polyclonal N-Cadherin 1:1000 (Biorbyt, Milan, Italy)). Cells were separately labelled with primary anbodies. Cross-reactivity of antibodies for the horse was previously tested in our laboratory on horse fibroblasts. Cells were then washed in PB2 (PB + 0.2% (w/v) BSA + 0.05 (w/v) % saponin) and incubated with goat anti-rabbit- FITC conjugated secondary antibody (Merck, Milan, Italy) 1:200 for 1 h. Nuclei were then labelled with Hoechst 33342 for fetal-MSCs and DAPI for adult-MSCs. The excess of secondary antibody and Hoechst/DAPI were removed by three washes with PB2. Images of MSCs were obtained with a Nikon Eclipse E400 microscope (Nikon Instruments Spa, Florence, Italy) using the software Nikon NIS-Elements.

### RT-PCR

At P3 10^5^ cells were snap-frozen and RNA was extracted using Nucleo Spin® RNA kit (Macherey-Nagel) following the manufacturer’s instructions. cDNAs were synthesized by RevertAid RT Kit (ThermoFisher Scientific) and used directly in PCR reactions, following the instructions of Maxima Hot Start PCR Master Mix (ThermoFisher Scientific). Expression of genes coding for MSC markers (CD90 and CD73), hematopoietic markers (CD34 and CD45), major histocompatibility complex (MHC) markers (MHC1 and MHC2), pro- and anti-inflammatory cytokines (TNFα, IL8, INFγ, IL4, ILβ1, IL6) and pluripotency markers (OCT4, SOX2, NANOG) were investigated. GAPDH was used as housekeeping gene to ensure proper expression of samples. Primers used are listed in Table 2. PCR products were visualized with ethidium bromide on a 2% (w/v) agarose gel.

**Table 2:** Sequence, source, amplicon size of primers used for PCR analysis. Primers are grouped in different categories

Primers	References	Sequences (5′ → 3′)	bp
Mesenchymal markers			
CD90	[52]	FW: TGCGAACTCCGCCTCTCT	93
		RW: GCTTATGCCCTCGCACTTG	
CD73	[52]	FW: GGGATTGTTGGATACACTTCAAAAG RW: GCTGCAACGCAGTGATTTCA	90
Hematopoietic markers			
CD34	[52]	FW: CACTAAACCCTCTACATCATTTTCTCCTA	101
		RW: GGCAGATACCTTGAGTCAATTTCA	
CD45	[52]	FW: TGATTCCCAGAAATGACCATGTA	101
		RW: ACATTTTGGGCTTGTCCTGTAAC	
Major histocompatibility complex markers			
MHC1	[53]	FW: GGAGAGGAGCAGAGATACA	218
		RW: CTGTCACTGTTTGCAGTCT	
MHC2	[53]	FW: TCTACACCTGCCAAGTG	178
		RW: CCACCATGCCCTTTCTG	
Pro- and anti-inflammatory cytokines			
TNFα	[54]	FW: GCTCCAGACGGTGCTTGTG	95
		RW: GCCGATCACCCCAAAGTG	
IL8	[54]	FW: CGGTGCCAGTGCATCAAG	81
		RW: TGGCCCACTCTCAATCACTCT	
INFγ	[55]	FW: GTGTGCGATTTTGGGTTCTTCTA	235
		RW: TTGAATGACCTGGTTATCT	
IL4	[55]	FW: CAACTTCATCCAGGGATGCAA	107
		RW: CAGTCAGCTCCATGCACGAAT	
ILβ1	[55]	FW: GAGGCAGCCATGGCAGCAGTA	257
		RW: TGTGAGCAGGGAACGGGTATCTT	
IL6	[56]	FW: AAACCACCTCAAATGGACCACTA	91
		RW: TTTTTCAGGGCAGAGATTTTGC	
Pluripotency markers			
OCT4	[36]	FW: TCCCAGGACATCAAAGCTCTGCAGA	679
		RW: TCAGTTTGAATGCATGGGAGAAGCCCAGA	
NANOG	[36]	FW: GACAGCCCCGATTCATCCACCAG	492
		RW: GCACCAGGTCTGACTGTTCCAGG	
SOX2	[36]	FW: GGCGGCAACCAGAAGAACAG	663
		RW: AGAAGAGGTAACCACGGGGG	
Housekeeping			
GAPDH	[36]	FW: GTCCATGCCATCACTGCCAC	262
		RW: CCTGCTTCACCACCTTCTTG	

### TEM

At P3 equine UCB, WJ, AT and BM-MSCs were fixed with 2.5% (v/v) glutaraldehyde in 0.1 M cacodylate buffer for 2 h at 4 °C and post fixed with a solution of 1% (w/v) osmium tetroxide in 0.1 M cacodylate buffer for 1 h at room temperature. Then, cells were embedded in epoxy resins after a graded-acetone serial dehydration step. The embedded samples were sectioned into ultrathin slices, stained with uranyl acetate and lead citrate solutions, and then observed by transmission electron microscope CM10 Philips (FEI Company, Eindhoven, The Netherlands) at an accelerating voltage of 80 kV. Images were recorded by Megaview III digital camera (FEI Company, Eindhoven, The Netherlands).

### Morphometric analysis

Morphometric analysis was carried out on 100 nm ultrathin slices by ITEM software (FEI Company, Eindhoven, The Netherlands). For cell area, 10 cell sections from each sample, from 5 to 6 randomly chosen regions, were acquired at 800 X and cell area was calculated for each cell and expressed as average value. For nuclear area, 10 cell sections from each sample, from 5 to 6 randomly chosen regions, were acquired at 3400X and the nuclear area was calculated in each section and expressed as average value. For the number of microvesicles, 10 cell sections from each sample, from 5 to 6 randomly chosen regions, were acquired at 19.000X and the number of microvesicles was calculated in each image and expressed as mean number of vesicles/field. For the diameter and area of microvesicles, from each sample, 20 randomly images of cytoplasm were acquired at 19000X and the diameter and area of the vesicle was calculated in each image and expressed as average value. For mitochondria number, 30 cell sections for each sample, from 5 to 6 randomly chosen regions, were acquired at 19.000X, calculated and the result was expressed as mean number of mitochondria/field. For mitochondrial area, 30 cell sections from each sample, from 5 to 6 randomly chosen regions, were acquired at 19.000X and mitochondrial area was calculated in each image and expressed as average value.

### Statistical analysis

Data are expressed as mean ± standard deviation (SD). Statistical analyses were performed using IBM SPSS Statistics 23 (IBM Corporation, Milan, Italy). Data were checked for normal distribution using a Shapiro-Wilk test, and then analysed using a one-way ANOVA or a Kruskal-Wallis Test. Bonferroni’s test was used for post hoc comparison. Student T-test was used for a further comparison of groups in the migration assay. For number of microvesicles and mitochondria, data were checked for Poisson distribution and then analysed using a Poisson regression. Significance was assessed for *P* < 0.05.

## Data Availability

The datasets used and/or analysed during the current study available from the corresponding author on reasonable request.

## Abbreviations

AA2P: 2-phospho-L-ascorbic acid trisodium salt
AT: Adipose tissue
BM: Bone marrow
CDs: Cell doublings
CT: Culture time
DMEM: Dulbecco’s modified Eagle medium
DPBS: Dulbecco’s phosphate buffered solution
DT: Doubling time
FBS: Foetal bovine serum
hTGFβ1: Human transforming growth factor β1
IBMX: 3-Isobutyl-1-methylxanthine
IF: Immunofluorescence
MHC: Major histocompatibility complex
MSCs: Mesenchymal stem cells
Nf: Final number
Ni: Initial number
P0: Passage 0
PB: Phosphate buffer
RT-PCR: Reverse transcriptase-polymerase chain reaction
SD: Standard deviation
TEM: Transmission electron microscopy
UCB: Umbilical cord blood
UCT: Umbilical cord tissue
WJ: Wharton’s jelly
α-SMA: α-smooth muscle actin

## References

[1] Kern S, Eichler H, Stoeve J, Klüter H, Bieback K (2006). Comparative analysis of mesenchymal stem cells from bone marrow, umbilical cord blood, or adipose tissue. Stem Cells.

[2] Montesinos JJ, Flores-Figueroa E, Castillo-Medina S, Flores-Guzmán P, Hernández-Estévez E, Fajardo-Orduña G, Orozco S, Mayani H (2009). Human mesenchymal stromal cells from adult and neonatal sources: comparative analysis of their morphology, immunophenotype, differentiation patterns and neural protein expression. Cytotherapy.

[3] Zhang X, Hirai M, Cantero S, Ciubotariu R, Dobrila L, Hirsh A, Igura K, Satoh H, Yokomi I, Nishimura T, Yamaguchi S, Yoshimura K, Rubinstein P, Takahashi TA (2011). Isolation and characterization of mesenchymal stem cells from human umbilical cord blood: reevaluation of critical factors for successful isolation and high ability to proliferate and differentiate to chondrocytes as compared to mesenchymal stem cells from bone marrow and adipose tissue. J Cell Biochem.

[4] Cavallo C, Cuomo C, Fantini S, Ricci F, Tazzari PL, Lucarelli E, Donati D, Facchini A, Lisignoli G, Fornasari PM, Grigolo B, Moroni L (2011). Comparison of alternative mesenchymal stem cell sources for cell banking and musculoskeletal advanced therapies. J Cell Biochem.

[5] Christodoulou I, Kolisis FN, Papaevangeliou D, Zoumpourlis V (2013). Comparative evaluation of human mesenchymal stem cells of fetal (Wharton's jelly) and adult (adipose tissue) origin during prolonged in vitro expansion: considerations for cytotherapy. Stem Cells Int.

[6] Toupadakis CA, Wong A, Genetos DC, Cheung WK, Borjesson DL, Ferraro GL, Galuppo LD, Leach JK, Owens SD, Yellowley CE (2010). Comparison of the osteogenic potential of equine mesenchymal stem cells from bone marrow, adipose tissue, umbilical cord blood, and umbilical cord tissue. Am J Vet Res.

[7] Burk J, Ribitsch I, Gittel C, Juelke H, Kasper C, Staszyk C, Brehm W (2013). Growth and differentiation characteristics of equine mesenchymal stromal cells derived from different sources. Vet J.

[8] Lange-Consiglio A, Corradetti B, Meucci A, Perego R, Bizzaro D, Cremonesi F (2013). Characteristics of equine mesenchymal stem cells derived from amnion and bone marrow: in vitro proliferative and multilineage potential assessment. Equine Vet J.

[9] Lange-Consiglio A, Tassan S, Corradetti B, Meucci A, Perego R, Bizzaro D, Cremonesi F (2013). Investigating the efficacy of amnion-derived compared with bone marrow-derived mesenchymal stromal cells in equine tendon and ligament injuries. Cytotherapy.

[10] Burk J, Gittel C, Heller S, Pfeiffer B, Paebst F, Ahrberg AB, Brehm W (2014). Gene expression of tendon markers in mesenchymal stromal cells derived from different sources. BMC Res Notes.

[11] Pratheesh MD, Dubey PK, Gade NE, Nath A, Sivanarayanan TB, Madhu DN, Somal A, Baiju I, Sreekumar TR, Gleeja VL, Bhatt IA, Chandra V, Amarpal SB, Saikumar G, Taru Sharma G (2017). Comparative study on characterization and wound healing potential of goat (*Capra hircus*) mesenchymal stem cells derived from fetal origin amniotic fluid and adult bone marrow. Res Vet Sci.

[12] Iacono E, Pascucci L, Rossi B, Bazzucchi C, Lanci A, Ceccoli M, Merlo B (2017). Ultrastructural characteristics and immune profile of equine MSCs from fetal adnexa. Reproduction.

[13] Iacono E, Brunori L, Pirrone A, Pagliaro PP, Ricci F, Tazzari PL, Merlo B (2012). Isolation, characterization and differentiation of mesenchymal stem cells from amniotic fluid, umbilical cord blood and Wharton's jelly in the horse. Reproduction.

[14] Li G, Zhang XA, Wang H, Wang X, Meng CL, Chan CY, Yew DT, Tsang KS, Li K, Tsai SN, Ngai SM, Han ZC, Lin MC, He ML, Kung HF (2009). Comparative proteomic analysis of mesenchymal stem cells derived from human bone marrow, umbilical cord, and placenta: implication in the migration. Proteomics.

[15] Wang W, Itaka K, Ohba S, Nishiyama N, Chung U, Yamasaki Y, Kataoka K (2009). 3D spheroid culture system on micropatterned substrates for improved differentiation efficiency of multipotent mesenchymal stem cells. Biomaterials.

[16] Kavanagh D, Robinson J, Kalia N (2014). Mesenchymal stem cell priming: fine-tuning adhesion and function. Stem Cell Rev.

[17] Sart S, Tsai A, Li Y, Ma T (2014). Three-dimensional aggregates of mesenchymal stem cells: cellular mechanisms, biological properties, and applications. Tissue Eng Part B Rev.

[18] Zeisberg M, Neilson EG (2009). Biomarkers for epithelial-mesenchymal transitions. J Clin Invest.

[19] Mazurkevych A, Malyuk M, Bezdieniezhnykh N, Starodub L, Kharkevych Y, Brusko E, Gryzińska M, Andrzej JA (2016). Immunophenotypic characterisation and cytogenetic analysis of mesenchymal stem cells from equine bone marrow and foal umbilical cords during in vitro culture. J Vet Res.

[20] Dominici M, Le Blanc K, Mueller I, Slaper-Cortenbach I, Marini F, Krause D, Deans R, Keating A, Prockop D, Horwitz E (2006). Minimal criteria for defining multipotent mesenchymal stromal cells. The International Society for Cellular Therapy position statement. Cytotherapy.

[21] Lin C, Ning H, Lin G, Lue T (2012). Is CD34 truly a negative marker for mesenchymal stromal cells?. Cytotherapy.

[22] Ranera B, Lyahyai J, Romero A, Vazquez FJ, Remacha AR, Bernal ML, Zaragoza P, Rodellar C, Martın-Burriel I (2011). Immunophenotype and gene expression profiles of cell surface markers of mesenchymal stem cells derived from equine bone marrow and adipose tissue. Vet Immunol Immunopathol.

[23] Braun J, Hack A, Weis-Klemm M, Conrad S, Treml S, Kohler K, Walliser U, Skutella T, Aicher WK (2010). Evaluation of the osteogenic and chondrogenic differentiation capacities of equine adipose tissue-derived mesenchymal stem cells. Am J Vet Res.

[24] Pascucci L, Curina G, Mercati F, Marini C, Dall’Aglio C, Paternesi B, Ceccarelli P (2011). Flow cytometric characterization of culture expanded multipotent mesenchymal stromal cells (MSCs) from horse adipose tissue: Towards the definition of minimal stemness criteria. Vet Immunol Immunopathol.

[25] De Schauwer C, Piepers S, van de Walle GR, Demeyere K, Hoogewijs MK, Govaere JLJ, Braeckmans K, van Soom A, Meyer E (2012). In search for cross-reactivity to immunophenotype equine mesenchymal stromal cells by multicolor flow cytometry. Cytometry Part A.

[26] Paebst F, Piehler D, Brehm W, Heller S, Schroeck C, Tárnok A, Burk J (2014). Comparative immunophenotyping of equine multipotent mesenchymal stromal cells: an approach toward a standardized definition. Cytometry A.

[27] De Schauwer C, Goossens K, Piepers S, Hoogewijs M, Govaere J, Smits K, Meyer E, Van Soom A, Van de Walle G (2014). Characterization and profiling of immunomodulatory genes of equine mesenchymal stromal cells from non-invasive sources. Stem Cells Res Ther.

[28] Lamalice L, Le Boeuf F, Huot J (2007). Endothelial cell migration during angiogenesis. Circ Res.

[29] György B, Szabò T, Pàsztòi M, Pàl Z, Mijàk P, Aradi B, Làszlò V, Pàllinger E, Pap E, Kittel A, Nagy G, Falus A, Buzás EI (2011). Membrane vesicles, current state-of-the-art: emerging role of extracellular vesicles. Cell Mol Life Sci.

[30] Miki T, Lehmann T, Cai H, Stolz DB, Strom SC (2005). Stem cell characteristics of amniotic epithelial cells. Stem Cells.

[31] Izumi M, Pazin BJ, Minervini CF, Gerlach J, Ross MA, Stolz DB, Turner ME, Thompson RL, Miki T (2009). Quantitative comparison of stem cell marker-positive cells in fetal and term human amnion. J Reprod Immunol.

[32] De Coppi P, Bartsch G, Siddiqui MM, Xu T, Santos CC, Perin L, Mostoslavsky G, Serre AC, Snyder EY, Yoo JJ, Furth ME, Soker S, Atala A (2007). Isolation of amniotic stem cell lines with potential for therapy. Nat Biotechnol.

[33] Pirjali T, Azarpira N, Ayatollahi M, Aghdaie MH, Geramizadeh B, Talai T (2013). Isolation and characterization of human mesenchymal stem cells derived from human umbilical cord Wharton’s jelly and amniotic membrane. Int J Organ Transplant Med.

[34] Shaer A, Azarpira N, Aghdaie MH, Esfandiari E (2014). Isolation and characterization of human mesenchymal stromal cells derived from placental deciduas basalis; umbilical cord Wharton’s jelly and amniotic membrane. Pak J Med Sci.

[35] Iacono E, Rossi B, Merlo B (2015). Stem cells from Foetal adnexa and fluid in domestic animals: an update on their features and clinical application. Reprod Dom Anim.

[36] Desmarais JA, Demers SP, Suzuki J, Laflamme S, Vincent P, Laverty S, Smith LC (2011). Trophoblast stem cell marker gene expression in inner cell mass-derived cells from parthenogenetic equine embryos. Reproduction.

[37] Warthemann R, Eildermann K, Debowski K, Behr R (2012). False-positive antibody signals for the pluripotency factor OCT4A (POU5F1) in testis-derived cells may lead to erroneous data and misinterpretations. Mol Hum Reprod.

[38] Guo CL, Liu L, Jia YD, Zhao XY, Zhou Q, Wang L (2012). A novel variant of Oct3/4 gene in mouse embryonic stem cells. Stem Cell Res.

[39] Hwang JY, Oh JN, Lee DK, Choi KH, Park CH, Lee CK (2015). Identification and differential expression patterns of porcine OCT4 variants. Reproduction.

[40] Liedtke S, Enczmann J, Waclawczyk S, Wernet P, Kögler G (2007). Oct4 and its pseudogenes confuse stem cell research. Cell Stem Cell.

[41] Teti G, Cavallo C, Grigolo B, Giannini S, Facchini A, Mazzotti A, Falconi M (2012). Ultrastructural analysis of human bone marrow mesenchymal stem cells during in vitro osteogenesis and chondrogenesis. Microsc Res Tech.

[42] Merlo B, Teti G, Mazzotti E, Ingrà L, Salvatore V, Buzzi M, Cerqueni G, Dicarlo M, Lanci A, Castagnetti C, Iacono E (2018). Wharton's jelly derived mesenchymal stem cells: comparing human and horse. Stem Cell Rev.

[43] García-Prat L, Martínez-Vicente M, Muñoz-Cánoves P (2016). Autophagy: a decisive process for stemness. Oncotarget.

[44] Sbrana FV, Cortini M, Avnet S, Perut F, Columbaro M, De Milito A, Baldini N (2016). The role of autophagy in the maintenance of Stemness and differentiation of mesenchymal stem cells. Stem Cell Rev.

[45] Biancone L, Bruno S, Deregibus MC, Tetta C, Camussi G (2012). Therapeutic potential of mesenchymal stem cell-derived microvesicles. Nephrol Dial Transplant.

[46] Suldina Lyubov A., Morozova Ksenia N., Menzorov Aleksei G., Kizilova Elena A., Kiseleva Elena (2018). Mitochondria structural reorganization during mouse embryonic stem cell derivation. Protoplasma.

[47] Sheng ZH (2017). The interplay of axonal energy homeostasis and mitochondrial trafficking and anchoring. Trends Cell Biol.

[48] Devine MJ, Kittler JT (2018). Mitochondria at the neuronal presynapse in health and disease. Nat Rev Neurosci.

[49] Rainaldi G, Pinto B, Piras A, Vatteroni L, Simi S, Citti L (1991). Reduction of proliferative heterogeneity of CHEF18 Chinese hamster cell line during the progression toward tumorigenicity. In Vitro Cell Dev Biol.

[50] Bellotti C, Duchi S, Bevilacqua A, Lucarelli E, Piccinini F (2016). Long term morphological characterization of mesenchymal stromal cells 3D spheroids built with a rapid method based on entry-level equipment. Cytotechnology.

[51] Lovati AB, Corradetti B, Cremonesi F, Bizzaro D, Consiglio AL (2012). Tenogenic differentiation of equine mesenchymal progenitor cells under indirect co-culture. Int J Artif Organs.

[52] Mohanty N, Gulati BR, Kumar R, Gera S, Kumar P, Somasundaram RK, Kumar S (2014). Immunophenotypic characterization and tenogenic differentiation of mesenchymal stromal cells isolated from equine umbilical cord blood. In Vitro Cell Dev Biol Anim.

[53] Corradetti B, Lange-Consiglio A, Barucca M, Cremonesi F, Bizzaro D (2011). Size-sieved subpopulations of mesenchymal stem cells from intervascular and perivascular equine umbilical cord matrix. Cell Prolif.

[54] Jischa S, Walter I, Nowotny N, Palm F, Budik S, Kolodziejek J, Aurich C (2008). Uterine involution and endometrial function in postpartum pony mares. Am J Vet Res.

[55] Castagnetti C, Mariella J, Pirrone A, Cinotti S, Mari G, Peli A (2012). Expression of interleukin-1 β, interleukin-8, and interferon-γ in blood samples obtained from healthy and sick neonatal foals. Am J Vet Res.

[56] Visser M, Pollitt C (2011). Lamellar leukocyte infiltration and involvement of IL-6 during oligofructose-induced equine laminitis development. Vet Immunol Immunopathol.

